# Multimodal evaluation of the bloodstream alteration before and after combined revascularization for Moyamoya disease

**DOI:** 10.3389/fneur.2023.1249914

**Published:** 2023-09-15

**Authors:** Lei Cao, Xiaoli Yuan, Yang Dong, Zeming Wang, Mengguo Guo, Dongpeng Li, Manxia Zhang, Dongming Yan, Bo Yang, Hongwei Li

**Affiliations:** ^1^Department of Neurosurgery, The First Affiliated Hospital of Zhengzhou University, Zhengzhou, China; ^2^Department of Hematology, Henan Provincial People’s Hospital, People’s Hospital of Zhengzhou University, Zhengzhou, Henan, China

**Keywords:** Moyamoya disease, perioperative hemodynamics, FLOW800, color Doppler, indocyanine green

## Abstract

**Objective:**

This study aimed to explore the hemodynamic changes before and after anastomosis in patients with Moyamoya disease (MMD) using multiple models.

**Methods:**

We prospectively enrolled 42 MMD patients who underwent combined revascularization. Intraoperative FLOW800 was performed before and after anastomosis, and parameters was collected, including maximum intensity, delay time, rise time, slope, blood flow index, and microvascular transit time (MVTT). Additionally, preoperative and postoperative hemodynamic parameters were measured using color Doppler ultrasonography (CDUS), including peak systolic velocity, end-diastolic velocity, resistance index (RI), pulsatility index (PI), and flow volume. Subsequently, the correlation between FLOW800 and CDUS parameters was explored.

**Results:**

A total of 42 participants took part with an average age of 46.5 years, consisting of 19 men and 23 women. The analysis of FLOW800 indicated that both the delay time and rise time experienced a substantial decrease in both the recipient artery and vein. Additionally, the MVTT was found to be significantly reduced after the surgery (5.7 ± 2.2 s vs. 4.9 ± 1.6, *p* = 0.021). However, no statistically significant differences were observed among the other parameters. Similarly, all postoperative parameters in CDUS hemodynamics exhibited significant alterations in comparison to the preoperative values. The correlation analysis between FLOW800 and CDUS parameters indicated a significant association between MVTT and RI and PI, no significant relationships were found among the other parameters in the two groups.

**Conclusion:**

The hemodynamic outcomes of the donor and recipient arteries demonstrated significant changes following bypass surgery. The parameter of time appears to be more precise and sensitive in assessing hemodynamics using FLOW800. Multiple evaluations of hemodynamics could offer substantial evidence for perioperative management.

## 1. Introduction

Moyamoya disease (MMD) is a chronic cerebral vascular disease (1). The pathology remains unclear (2). Revascularization surgery could reduce the risk of cerebral infarction and improve the long-term prognosis of neurocognitive function (3). Surgical procedures include direct revascularization, indirect revascularization and combined revascularization. Superficial temporal artery-middle cerebral artery (STA-MCA) anastomosis is the most common direct revascularization bypass, while indirect revascularization surgeries such as encephalo-arterio-synangiosis (EDAS), encephalo-myo-synangiosis, encephalo-duro-synangiosis and multiple burr hole surgery are widely accepted. Previous studies have shown Improvements in cerebral hemodynamics after revascularization bypass (4). However, the assessment of perioperative cerebral hemodynamics has not been clearly understood.

FLOW800 is a specialized software that utilizes indocyanine green (ICG) videoangiography to analyze data and generate color delay mapping (5, 6). This new modality, ICG-FLOW800, allows for the semi-quantitative evaluation of cerebral blood flow changes (7). It provides direct regional hemodynamic parameters, which can guide immediate intraoperative decisions. Additionally, color Doppler ultrasonography (CDUS) can be used as a non-invasive tool to quantitatively monitor blood flow changes. Several reports have detected significant hemodynamic changes in the donor superficial temporal artery (STA) after different revascularization bypass procedures using CDUS (8–12).

In this study, we used ICG-FLOW800 video angiography mapping method to assess the cerebral perfusion changes before and after anastomosis, and we used CDUS to monitor the hemodynamic changes of STA trunk preoperatively and postoperatively. This study aimed to access perioperative blood flow changes of donor STA, and evaluate cerebral hemodynamics of recipient artery before and after anastomosis.

## 2. Materials and methods

### 2.1. Patients and management

We prospectively investigated 42 patients from August 1st, 2022 to July 28th, 2023. According to the diagnostic criteria (13), all patients were diagnosed as MMD and underwent STA-MCA direct bypass combined EDAS indirect bypass. After combined revascularization procedure, fluid therapy was given and blood pressure was maintained at an appropriate level to ensure enough circulation volume. Each patient underwent magnetic resonance imaging (MRI), cerebral perfusion imaging and DSA preoperatively, computed tomography was performed routinely within 24 h after the operation, additional MRI was performed when new symptoms appeared after surgery.

All of the patients met the Chinese guidelines for the diagnosis and treatment of MMD and moyamoya syndrome set by the Stroke Prevention Project Committee, National Health and Family Planning Commission, China. This study was approved by the Ethics Committee of The First Affiliated Hospital of Zhengzhou University.

### 2.2. Surgical procedure

For patients, a combined revascularization surgery involving a direct STA-MCA bypass and an indirect EDAS bypass was conducted. The frontal and parietal branches of the STA were isolated from the scalp flap and utilized as donor arteries. The anastomosis between one branch of the STA and the MCA was performed in an end-to-side fashion under a micromanipulation microscope. The choice of the recipient artery, based on criteria such as reduced cerebral blood flow (CBF) region, vessel diameter and donor accessibility, was made under the microscope. Simultaneously, the other branch of the STA was positioned onto the brain surface. Subsequently, the bone flap was replaced and the facial tissue layered with sutures. All combined revascularization surgeries were performed by the same surgeon.

### 2.3. ICG-FLOW800 analysis

**Figure 1:**
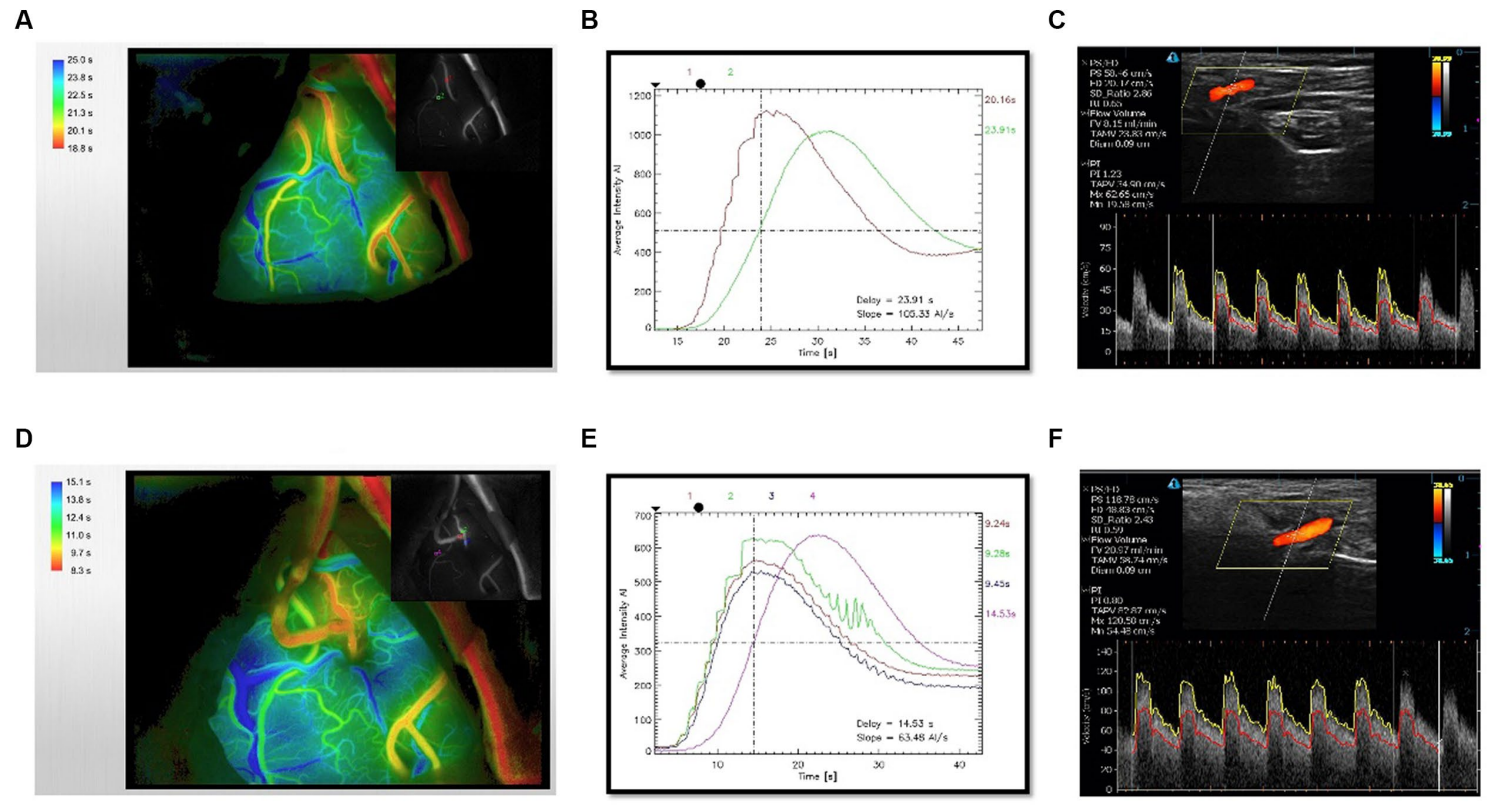
Evaluation of hemodynamics before and after bypass. **(A)** Time-delayed color map of hemodynamics provided by ICG-FLOW800 before anastomosis. **(B)** Intensity-time curve provided by FLOW800 before anastomosis. **(C)** Preoperative CDUS imaging of STA trunk. **(D)** Time-delayed color map of hemodynamics provided by ICG-FLOW800 after anastomosis. **(E)** Intensity-time curve provided by FLOW800 after anastomosis. **(F)** Postoperative CDUS imaging within 24 h. The color map is color-coded according to the order of the ICG contrast agent flowing through the parts and the length of the ICG contrast agent, the earliest passage area was indicated by red color.

To assess the cerebral hemodynamics before and after bypass around anastomotic site, intraoperative ICG video was analyzed using the FLOW800 software (Zeiss, Germany). 25 mg of ICG was dissolved in 10 mL of normal saline in each bolus. No adverse reactions to ICG occurred in patients. At each angiography, ICG was injected into the cubital vein and fluoroscopy was performed. According to previous report (5), we did not attempt to control a high degree of standardization of the angiography condition before (Figures 1A,B) and after the anastomosis (Figures 1D,E), for example, multiples, angle, focal length and magnification, and distance from the field. Because such an approach would not be applied broadly on a routine surgical procedure. Regions of interest (ROI) were set at more than 2 points on the brain surface in the surgical area. The same region of ROIs was selected before and after the anastomosis.

The intensities of ICG transit curves were recorded and the data from ROIs could be exported. Hemodynamic parameters were calculated from the selected points, including maximum intensity (arbitrary intensity, AI), delay time (second, s), rise time (second, s), slope (AI/s), blood flow index (BFI, AI/s), and the microvascular transit time (MVTT, s). Delay time was defined as the time interval between 0 and 50% of maximum fluorescence intensity. Rise time was defined as the time interval between 10 and 90% of the maximum fluorescence intensity (14). The cerebral BFI was calculated as ratio of difference in fluorescence intensity and rise time (7). MVTT was calculated as venous T_1/2_ peak − arterial T_1/2_ peak considering the peak intensity lasts for several seconds (15).

### 2.4. CDUS assessment

Hemodynamics of donor STA were measured using CDUS device (Tensor3300) preoperatively (Figure 1C) and within 24 h postoperatively (Figure 1F). The following six valuable hemodynamic parameters were selected: peak systolic velocity (PSV), end-diastolic velocity (EDV), ratio of PSV and EDV (SD), resistance index (RI), pulsatility index (PI), and flow volume (FV). SD was defined as the ratio of PSV and EDV, RI was calculated based on the formula: RI = (PSV−EDV)/PSV, PI was calculated based on the formula: PI = (peak systole − end-diastole)/ time-averaged mean maximal velocity (TAMX). The RI and PI value reflect the vascular resistance to blood flow (16). CDUS examinations were performed by the same experienced technician. All patients were relaxed in the supine position with their head turned to the side. The hook-shaped probe was placed on the STA trunk to measure the blood flow and the same parameters were measured before and after bypass surgery. The examination was illustrated in Figure 1.

### 2.5. Statistical analysis

Categorical variable data were described by frequency and ratio. Numerical variable data were shown as mean ± standard deviation. Fisher’s exact test for categorical variables and a paired sample *t* test for numerical variables were applied to data generated before and after operation. Statistical analysis was performed using SPSS 21.0, a significance level of *p* < 0.05 was employed to determine statistical significance.

## 3. Results

### 3.1. Clinical data

In this research, a total of 42 candidates were admitted. Among them, there were 19 men and 23 women, with an average age of 46.5 ± 9 years (range, 27–58 years). The majority of the patients, 39 out of 42, presented with symptoms caused by cerebral ischemia. Specifically, 23 patients complained of headache and dizziness, 7 patients reported limb numbness, 5 patients experienced speech disorder, 3 patients had blurred vision, and 1 patient had cognitive impairment. The remaining 3 patients were diagnosed with intraventricular hemorrhage. Intraoperative assessments confirmed the patency of the bypass in all patients. The demographic and clinical characteristics of the patients can be found in Table 1.

**Table 1:** Demographics of the enrolled patients (*N* = 42).

Parameters	Patients counts
Sex	
Male	19 (45.2%)
Female	23 (54.8%)
Clinical type	
Ischemic	39 (92.9%)
Hemorrhagic	3 (7.1%)
Surgery side	
Left	19 (45.2%)
Right	23 (54.8%)
Mean age (years)	46.5 ± 9
Clinical symptoms	
headache and dizziness	23 (54.8%)
Limb numbness	7 (16.7%)
Speech disorder	5 (11.9%)
Blurred vision	3 (7.1%)
Cognitive impairment	1 (2.3%)

### 3.2. Results of ICG-FLOW800 analysis

**Figure 2:**
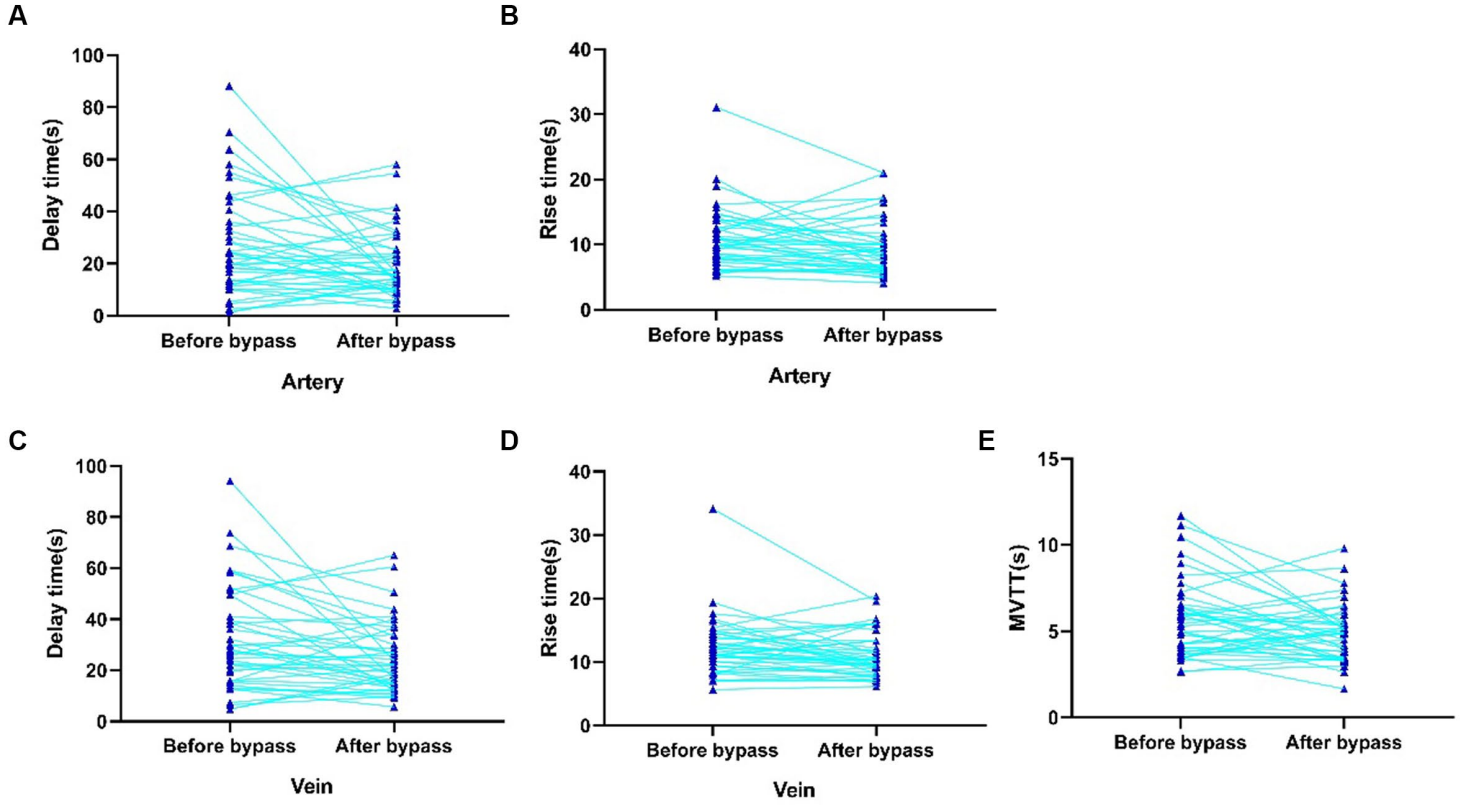
Significantly changed intraoperative hemodynamic parameters of recipient artery and vein detected by FLOW800. **(A)** Delay time of recipient artery before anastomosis. **(B)** Rise time of recipient artery after anastomosis. **(C)** Delay time of vein before anastomosis. **(D)** Rise time of vein after anastomosis. **(E)** MVTT before and after anastomosis.

We investigated the cortical perfusion by using ICG videoangiography analyzed with FLOW 800 software. Parameters before and after anastomosis were detected in both artery and vein. Hemodynamic values in the recipient artery were as follows: maximum intensity (538.3 ± 199.2 AI vs. 541.6 ± 238.8 AI), slope (58.8 ± 36.9 AI/s vs. 63.6 ± 36.1 AI/s), and blood flow index (45.1 ± 22.8 AI/s vs. 47.3 ± 30.7 AI/s). There was no significant difference in maximum intensity, slope, and blood flow index. However, there was a significant reduction in delay time (26.8 ± 19.9 s vs. 19.6 ± 12.6 s, *p* = 0.018) and rise time (11.1 ± 4.8 s vs. 9.4 ± 4.3 s, *p* = 0.018) after the anastomosis surgery (Figures 2A,B). In the vein, the hemodynamic values were as follows: maximum intensity (502.85 ± 199.8 AI vs. 524.7 ± 166.8 AI), slope (42.4 ± 25.4 AI/s vs. 47.5 ± 26.4 AI/s), and blood flow index (45.1 ± 25.1 AI/s vs. 53.8 ± 27.69 AI/s) (Figures 2C,D). Similar to the artery, there was no significant difference in maximum intensity, slope, and blood flow index. However, there was a significant reduction in delay time (31.8 ± 20.2 s vs. 24.8 ± 13.7 s, *p* = 0.014) and rise time (12.3 ± 4.6 s vs. 11.1 ± 3.5 s, *p* = 0.043) after the anastomosis surgery. Furthermore, the MVTT was significantly reduced after the bypass surgery (5.7 ± 2.2 s vs. 4.9 ± 1.6 s, *p* = 0.021) (Figure 2E). More detailed results can be found in Supplementary Table S1; Figure 2.

### 3.3. Results of CDUS evaluation

**Figure 3:**
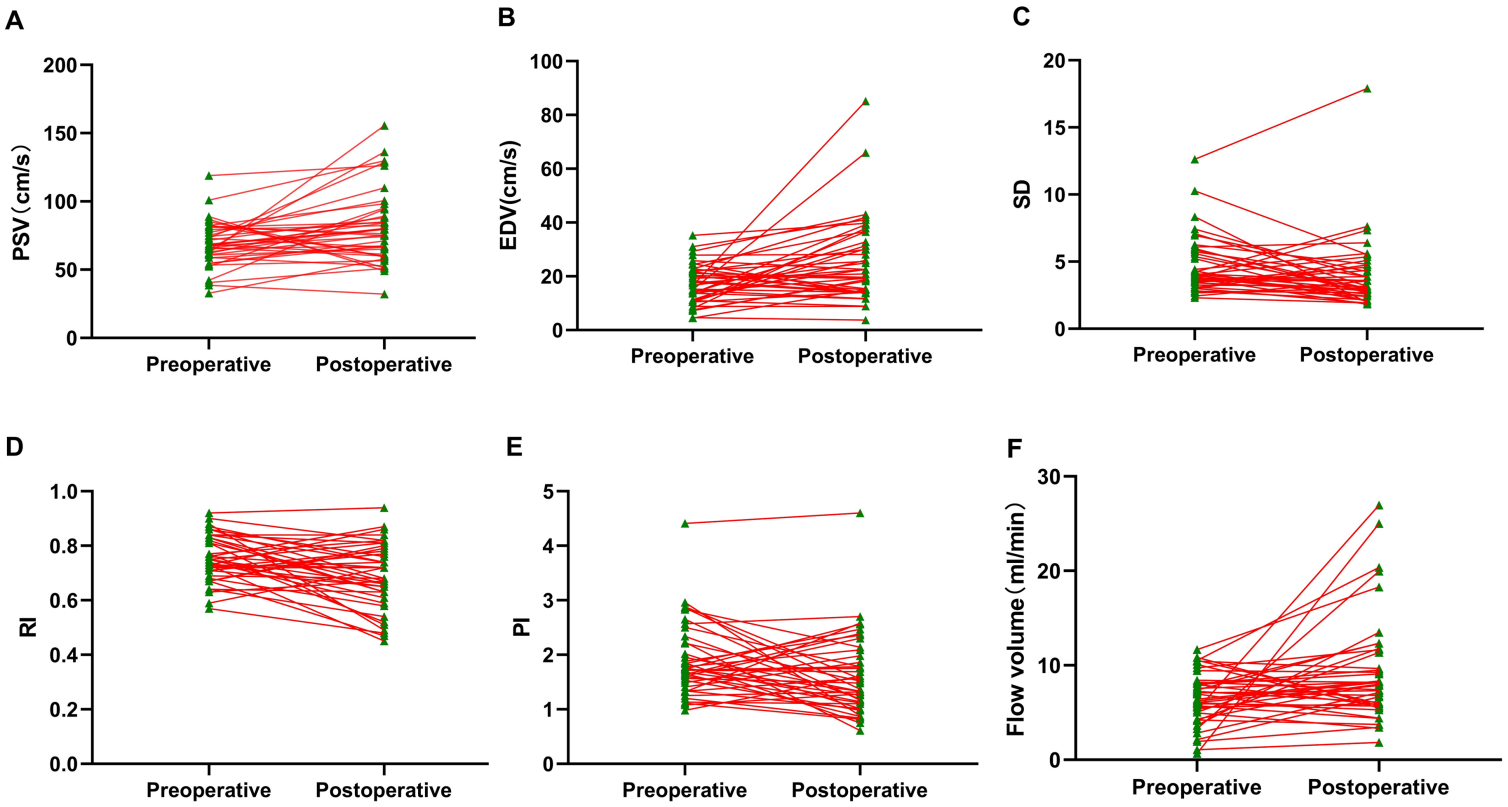
Perioperative hemodynamics of STA trunk detected by CDUS. **(A)** Parameter of PSV. **(B)** Parameter of EDV. **(C)** Parameter of SD. **(D)** Parameter of RI. **(E)** Parameter of PI. **(F)** Parameter of flow volume. Compared with preoperative values, all the postoperative hemodynamic parameters within 24 h were significantly changed.

We then investigated the hemodynamic changes in the donor STA before and after the operation. The postoperative examination revealed that the average PSV and EDV in the STA increased from 68.57 cm/s and 17.12 cm/s to 80.55 cm/s and 26.07 cm/s, respectively (Figures 3A–C). The preoperative values of RI and PI in the STA were 0.75 and 1.87, respectively, while the postoperative values decreased to 0.68 and 1.60 (Figures 3D,E). Moreover, there was a significant increase in the mean PSV and EDV within the first 24 h after the operation, and the reduction in RI and PI between the preoperative and postoperative values was highly significant. Additionally, the mean flow volume in the STA was 6.52 mL/min preoperatively (Figure 3F), and it increased to 9.27 mL/min after the combined surgery. For further details, please refer to Supplementary Table S2 and Figure 3.

**Figure 4:**
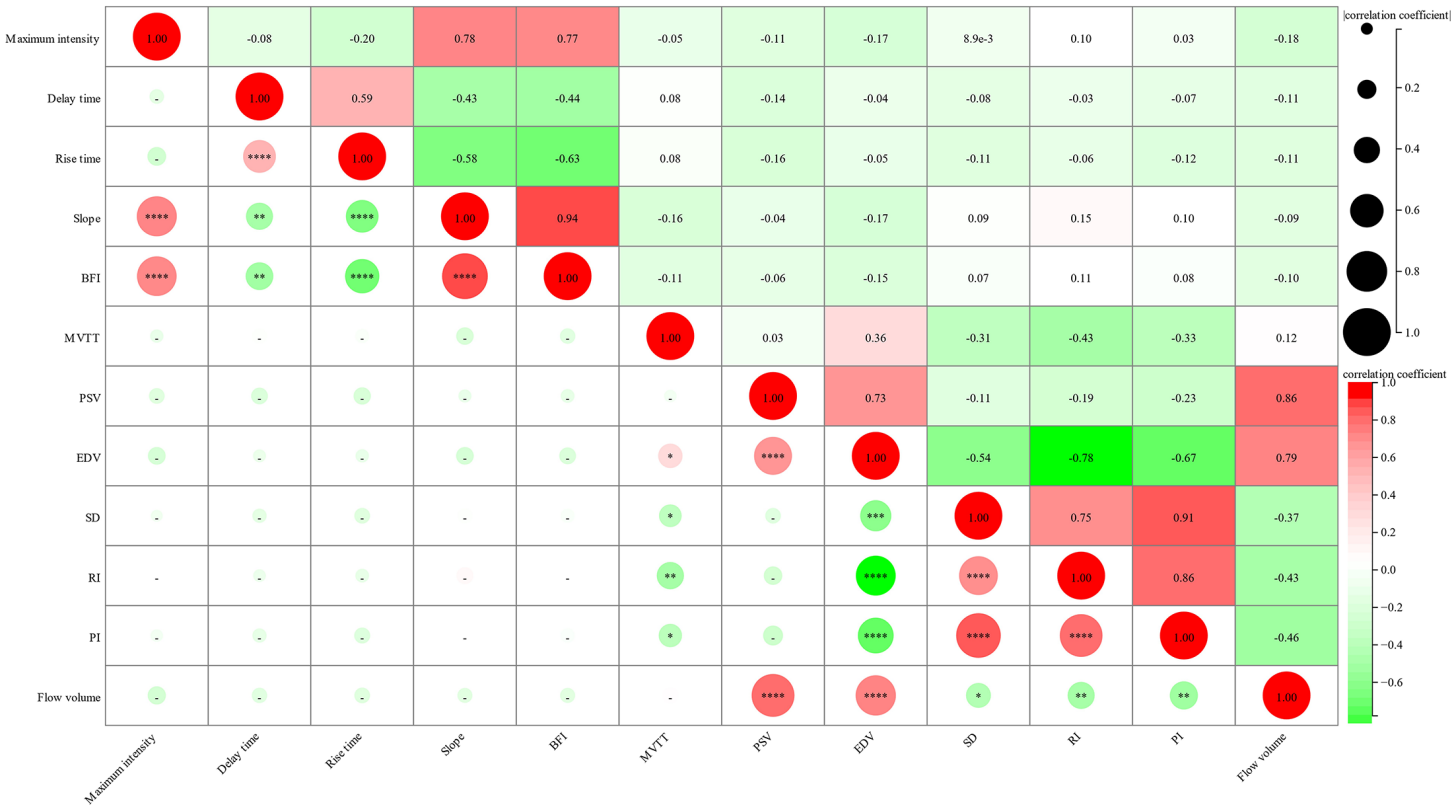
Correlation between intraoperative FLOW800 parameters of recipient artery and postoperative hemodynamic parameters of donor STA. The size of circle represents the correlation coefficient, the gradient color represents the *p*-value.

Furthermore, we investigated the relationship between cortical perfusion detected by FLOW800 and the hemodynamic parameters measured using CDUS. The correlation between six parameters of recipient artery after anastomosis and six postoperative parameters of donor STA was analyzed. Notably, MVTT showed a significant negative correlation with RI and PI and positive correlation with EDV. However, no significant correlations were detected among the other parameters between the two groups, including the FLOW800-specific parameter BFI and the FV of the donor STA (Figure 4).

## 4. Discussion

Revascularization is an effective treatment for MMD. As intracranial vascular and the extracranial vascular belong to different vascular systems, revascularization leads to immediate changes in hemodynamics. The postoperative SPECT has presented the typical CBF improvement pattern with temporary local hyperperfusion on the first day after surgery, followed by a spread of CBF to a larger vascular area (17). Thus, it is crucial to assess these changes using different techniques due to their close relationship with cerebral hyperperfusion syndrome (CHS) (18–20), which has a significant impact on perioperative management. In this study, multimodal assessment of perioperative hemodynamics was performed, and the relationship between FLOW800 values and CDUS parameters detected after the bypass surgery was explored.

FLOW800 has the potential to visualize hemodynamic changes post intracranial and extracranial bypass procedures. By evaluating the ICG fluorescein angiography video, FLOW800 provides a visually time-delayed color map and data analysis of hemodynamics. This color map can assist in selecting the appropriate recipient artery and guiding immediate intraoperative decisions. Additionally, FLOW800 enables the evaluation of local hemodynamic characteristics intraoperatively. Prior studies have reported a decrease in circulation time of corresponding branches and an increase in cortical perfusion (21). With FLOW800, provided direct perfusion range (22) and change rates of peak cerebral blood volume, regional cerebral blood flow, and time to peak (19) offer potential predictive value for CHS. STA-MCA bypass has been shown to significantly reduce MVTT[15]and improve BFI (23). Nonetheless, we observed no significant differences in parameters such as maximum intensity, slope, and BFI before and after bypass. This inconsistency, particularly in BFI results (23), could be attributed to the videoangiography procedure and the method of calculating rise time. In their study, 25 mg ICG was dissolved in 5 mL of water and the rise time was defined as time interval between 20 and 80% of maximum fluorescence intensity. Furthermore, numerous factors can influence these parameters, including injection speed, microscope distance, angulation, and individual patient differences. According to a report (5), parameters relying solely on fluorescence intensity are highly affected, while those based solely on time exhibit minimal variability, and parameters that consider both time and fluorescence level demonstrate intermediate variability. Moreover, FLOW800 is a semi-quantitative analysis software, it exhibits poor accuracy when comparing multiple cases, although an elevated trend has been observed. Therefore, FLOW800’s time parameter may offer greater accuracy and sensitivity in evaluating hemodynamics.

CDUS has been applied to evaluate the hemodynamics of donor STA and recipient arteries in different kinds of revascularizations. Postoperative and follow-up exams revealed a significantly higher mean PSV and EDV of STA (8, 11). Hemodynamic parameters of STA, maxillary artery and ophthalmic artery were exhibited statistically significant differences between compensatory group and non-compensatory group (10). The PI and RI at 1 week and 3 months after surgery were significantly lower than the preoperative values (12). In our previous report, a fluctuate hemodynamics of the postoperative STA blood flow has been detected using CDUS (24). In this study, all the hemodynamic parameters changed significantly within 24 h after surgery. Compared to preoperative values, there was a significant increase in PSV and EDV. As the STA and internal carotid artery belong to different vascular systems, postoperative RI and PI of the STA decreased significantly. Moreover, a substantial augmentation in the flow volume of the STA was observed, implying the elevated cerebral perfusion. The decreased RI and PI, increased flow volume in STA may be due to the low resistance of the intracranial vascular bed. The different pressure gradients were considered to be the main driving factor for the blood flow (22, 25). Therefore, these hemodynamic results suggest that combined revascularization is an effective treatment for MMD.

To further explore the relationship of blood flow hemodynamics between intraoperative FLOW800 parameters and postoperative CDUS hemodynamic values within 24 h, a correlation analysis was conducted. The results revealed that MVTT has a significant negative correlation with RI and PI. Prolonged MVTT in patients with MMD has been reported as compensatory mechanism for impaired hemodynamics (6, 26). As a chronic cerebrovascular disease, we hypothesized there existed an MMD-specific self-recirculation collateral vascular network in MMD patients and the work have not been published. The more developed the collateral vessels, the lower the resistance. It was well known that both RI and PI demonstrated a resistance of cerebral blood flow, and MVTT was derived as the time required for blood to flow from the arterial phase to the venous phase. Thus, a lower resistance indicates longer collateral vessel pathways and consequently a longer MVTT. Another interesting finding is the positive correlation between MVTT and EDV. In our previous study, we observed decreased RI, mainly caused by increased EDV, following combined revascularization (24). Therefore, it is plausible that this positive correlation also relates to the MMD-specific compensation mechanism and the self-recirculation collateral cerebrovascular network. However, the results did not show a direct and significant correlation among other parameters. On the one hand, it may be attributed to the semi-quantitative analysis of FLOW800. On the other hand, the intraoperative and postoperative hemodynamic parameters were assessed at different time points, despite CDUS being performed within 24 h after bypass surgery. Nevertheless, more research is needed to fully understand these relationships.

## 5. Conclusion

Collectively, the postoperative hemodynamics were significantly altered compared with that of pre-operation. FLOW800 and CDUS were alternative tools to evaluate blood flow in MMD. Multimodal evaluation of the hemodynamic changes before and after combined revascularization was useful and necessary for Moyamoya disease. It could provide critical evidence to perioperative management.

## Data availability statement

The original contributions presented in the study are included in the article/Supplementary material, further inquiries can be directed to the corresponding authors.

## Ethics statement

The studies involving humans were approved by Ethics Committee of The First Affiliated Hospital of Zhengzhou University. The studies were conducted in accordance with the local legislation and institutional requirements. The participants provided their written informed consent to participate in this study.

## Author contributions

LC, XY, and YD designed and wrote the manuscript. ZW, MG, and DL provided radiological resources and drafted the pictures. MZ and DY provided clinical resources. HL and BY revised it critically for intellectual content. All authors contributed to the article and approved the submitted version.

## Conflict of interest

The authors declare that the research was conducted in the absence of any commercial or financial relationships that could be construed as a potential conflict of interest.

## Publisher’s note

All claims expressed in this article are solely those of the authors and do not necessarily represent those of their affiliated organizations, or those of the publisher, the editors and the reviewers. Any product that may be evaluated in this article, or claim that may be made by its manufacturer, is not guaranteed or endorsed by the publisher.
