# Supplementary material for: Multimodal evaluation of the bloodstream alteration before and after combined revascularization for Moyamoya disease
Source: Front Neurol. 2023 Sep 15;14:1249914. doi: 10.3389/fneur.2023.1249914 (PMC10540193; doi:10.3389/fneur.2023.1249914)
Supplement: Supplementary file 1 [file Table_1.DOCX]

**Supplementary table 1.**

The FLOW800 values measured in brain surface before and after surgery

| Parameters | preoperative | postoperative | *P* value |
| --- | --- | --- | --- |
| Artery |  |  |  |
| Maximum intensity (AI) | 538.3±199.2 | 541.6±238.8 | 0.941 |
| Delay time (s) | 26.8±19.9 | 19.6±12.6 | 0.018 |
| Rise time (s) | 11.1±4.8 | 9.4±4.3 | 0.018 |
| Slope (AI/s) | 58.8±36.9 | 63.6±36.1 | 0.471 |
| BFI (AI/s) | 57.4±36.1 | 66.3±37.9 | 0.211 |
| Vein |  |  |  |
| Maximum intensity (AI) | 502.85±199.8 | 524.7±166.8 | 0.570 |
| Delay time (s) | 31.8±20.2 | 24.8±13.7 | 0.014 |
| Rise time (s) | 12.3±4.6 | 11.1±3.5 | 0.043 |
| Slope (AI/s) | 42.4±25.4 | 47.5±26.4 | 0.175 |
| Brain flow index (AI/s) | 45.1±25.1 | 53.8±27.69 | 0.054 |
| MVTT(s) | 5.7±2.2 | 4.9±1.6 | 0.021 |

**Supplementary table 2.**

The CDUS values measured in donor STA before and after surgery

| Parameters | preoperative | postoperative | *P* value |
| --- | --- | --- | --- |
| PSV (cm/s) | 68.57±16.25 | 80.55±25.91 | 0.004 |
| EDV (cm/s) | 17.12±7.22 | 26.07±15.60 | 0.001 |
| SD | 4.66±2.14 | 3.92±2.64 | 0.040 |
| RI | 0.75±0.08 | 0.68±0.12 | 0.001 |
| PI | 1.87±0.66 | 1.60±0.74 | 0.022 |
| FV (ml/min) | 6.52±2.74 | 9.27±5.56 | 0.006 |
